# An examination of the relationships between the neighborhood social environment, adiposity, and cardiometabolic disease risk in adolescence: a cross-sectional study

**DOI:** 10.1186/s12889-023-16580-0

**Published:** 2023-09-01

**Authors:** Kara D. Denstel, Robbie A. Beyl, Denise M. Danos, Maura M. Kepper, Amanda E. Staiano, Katherine T. Theall, Tung-Sung Tseng, Stephanie T. Broyles

**Affiliations:** 1 Pennington Biomedical Research Center, 6400 Perkins Rd, Baton Rouge, Baton Rouge, LA 70808 USA; 2 School of Public Health, Louisiana State University Health Sciences Center, New Orleans, LA USA; 3 Prevention Research Center, Washington University in St. Louis, One Brookings Drive, St. Louis, MO USA; 4 Department of Epidemiology and Department of Social, Behavioral, and Population Sciences, Tulane University School of Public Health and Tropical Medicine, New Orleans, LA USA

**Keywords:** Neighborhood social environment, Neighborhood disadvantage, Neighborhood disorder, Child obesity, Adolescent health

## Abstract

**Background:**

Disadvantaged neighborhood environments are a source of chronic stress which undermines optimal adolescent health. This study investigated relationships between the neighborhood social environment, specifically, chronic stress exposures, adiposity, and cardiometabolic disease risk factors among 288 Louisiana adolescents aged 10 to 16 years.

**Methods:**

This cross-sectional study utilized baseline data from the Translational Investigation of Growth and Everyday Routines in Kids (TIGER Kids) study. Adolescent data were obtained using self-reported questionnaires (demographics and perceived neighborhood disorder), anthropometry, body imaging, and a blood draw while objective neighborhood data for the concentrated disadvantage index were acquired from the 2016 American Community Survey five-year block group estimates, 2012–2016. Multilevel linear regression models were used to examine whether neighborhood concentrated disadvantage index and perceived neighborhood disorder were associated with body mass index, waist circumference, body fat, adipose tissue, blood pressure, and lipids. We performed multilevel logistic regression to determine the odds of elevated adiposity and cardiometabolic disease risk for adolescents living in neighborhoods with varying levels of neighborhood concentrated disadvantage and disorder.

**Results:**

Adolescents living in neighborhoods with higher disadvantage or disorder had greater waist circumference and total percent body fat compared to those in less disadvantaged and disordered neighborhoods (*p* for trend < 0.05). Neighborhood disadvantage was also positively associated with percentage of the 95^th^ Body Mass Index percentile and visceral abdominal adipose tissue mass while greater perceived neighborhood disorder was related to higher trunk fat mass and diastolic blood pressure (*p* for trend < 0.05). Living in the most disadvantaged was associated with greater odds of obesity (OR: 2.9, 95% CI:1.3, 6.5) and being in the top tertile of body fat mass (OR: 3.0, 95% CI: 1.4, 6.6). Similar results were found with neighborhood disorder for odds of obesity (OR: 2.1, 95% CI:1.1, 4.2) and top tertile of body fat mass (OR: 2.1, 95% CI:1.04, 4.1).

**Conclusions:**

Neighborhood social environment measures of chronic stress exposure were associated with excess adiposity during adolescence, and relationships were most consistently identified among adolescents living in the most disadvantaged and disordered neighborhoods. Future studies should account for the influences of the neighborhood environment to stimulate equitable improvements in adolescent health.

**Clinical Trials Registration:**

# NCT02784509.

## Introduction

Obesity prevention during childhood and adolescence is a primary focus of many public health campaigns in the United States (U.S.). Twenty-six percent of U.S. adolescents 12 to 19 years of age have obesity according to the 2017–2020 (pre-pandemic) National Health and Nutrition Examination Survey, which represents a nearly four percentage point rise in obesity prevalence from 2015–2016 [1]. Additionally, the prevalence of severe obesity (body mass index, BMI, at or above 120% of the 95^th^ percentile) among adolescents nearly tripled from 2.6% in 1988–1992 [2] to 7.7% in 2015–2016 [3]. Moreover, obesity and excess adiposity during adolescence predict the development of other cardiometabolic diseases at younger ages [4]. As such, nearly one in five U.S. adolescents has prediabetes [5] and 10% have elevated blood pressure [6], which increase the risk of progressing to type 2 diabetes and hypertension, respectively [6].

Adolescents who live in neighborhoods where they are exposed to chronic stressors, such as crime and violence, overcrowding, noise, and food insecurity, which we term ‘disadvantaged neighborhoods,’ may experience impaired behavioral, psychological and physical development [7]. The body’s physiologic stress response is well-adapted for acute stressors (i.e., ‘fight or flight’ from a time-limited threat) but ill-suited for responding to modern chronic stressors. Long-term stress exposure can result in sustained activation and elevation of stress hormones which increase inflammation, elevate blood pressure, mobilize glucose, slow digestion, and trigger abdominal fat deposition [8]. The sustained activation of the stress response may result in disequilibrium of the hypothalamic–pituitary–adrenal (HPA) axis and other physiologic functions which underlie the development of stress-related diseases. In addition to frequent increases in blood pressure that may accelerate heart disease, lack of sufficient deactivation of the stress response may cause overexposure to stress response chemicals, such as glucocorticoids and catecholamines, leading to systemic inflammation, increased fat storage, and the development of obesity and type 2 diabetes [9]. As shown by Pervanidou, [10] chronic stress exposure is linked to poor cardiometabolic health through both these biologic pathways as well as behavioral pathways. Stress exposure promotes negative behaviors, including emotional eating, preference for calorie-dense, nutrient-poor “comfort foods”, sedentary behaviors, and a lack of adequate sleep which are also risk factors for obesity, hypertension, and metabolic disease [10]. While resilience and positive stress coping skills may help ameliorate some of these negative health effects, living in chronically disadvantaged neighborhoods hinders the development of those healthy behaviors due to the lack of adequate opportunities for safe physical activity and access to fresh, nutrient-rich foods [11].

While cardiometabolic disease is more prevalent among adults, it is essential to better understand how neighborhood social environment measures of chronic stress exposure relate to adiposity and cardiometabolic health during adolescence. According to the Life Course Perspective, adolescence is a critical period during which adverse exposures accumulate and have the most significant impact on future health [12, 13]. Indeed, researchers found that neighborhood deprivation exposure during adolescence (ages 10 through 17) was more predictive of adult obesity compared to exposure earlier in childhood (birth to age nine) [14]. While this field of research has grown within adolescent populations, it has primarily focused on anthropometric measures of adiposity (e.g., BMI and waist circumference) [15–21]. A recent review [16] concluded that neighborhood deprivation is consistently related to higher BMI and the probability of obesity among adolescents; however, it is unclear how these findings relate to more precise metrics of adiposity, including body fat and adipose tissue mass, or other cardiometabolic disease risk factors. The present study investigated relationships between the neighborhood social environment (specifically, chronic stress exposures measured as neighborhood concentrated disadvantage index and perceived neighborhood disorder) and a comprehensive set of adiposity metrics and cardiometabolic disease risk factors, including lipids, blood pressure, and a composite cardiometabolic disease risk score.

## Methods

### Study design and participants

This cross-sectional study utilized baseline data from the Translational Investigation of Growth and Everyday Routines in Kids (TIGER Kids; NCT02784509) prospective cohort study, which included 342 adolescents aged 10 to 16 years of which approximately 50% were overweight or had obesity or severe obesity. The study was approved by the Pennington Biomedical Institutional Review Board, and data were collected from June 2016 to August 2018. Adolescents were recruited using a list of previous study participants, social media advertisements, and through schools and community groups in greater Baton Rouge, Louisiana. Eligible study participants and a caregiver participated in informed assent and consent procedures prior to enrollment. Inclusion criteria included being aged 10–16 years, having a body weight less than 500 pounds due to equipment limitations, and the ability to understand and complete study procedures. Adolescents were excluded from TIGER Kids participation if they were pregnant, on a restrictive diet, or had a significant physical or mental disability that impeded physical activity. Full eligibility criteria are published elsewhere [22]. The TIGER Kids study measurements relevant to the present study were anthropometry, body composition imaging, a blood draw, and parent- and adolescent-reported surveys collected using REDCap (Research Electronic Data Capture) [23, 24].

**Fig. 1:**
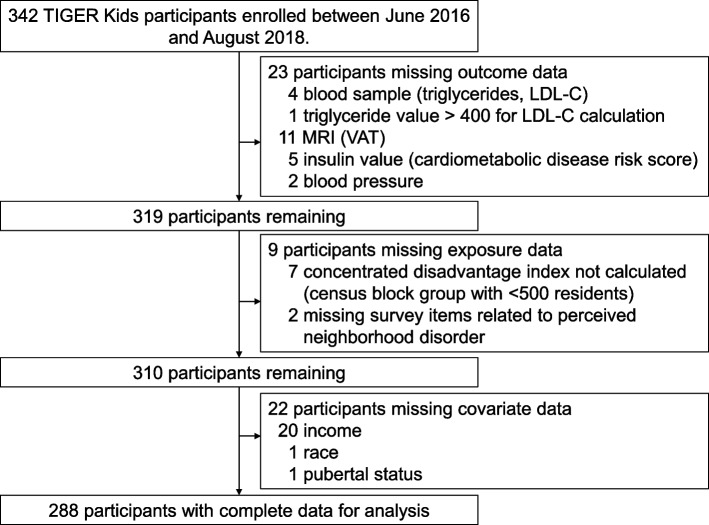
The flow of missing data from the parent TIGER Kids study to the analytic sample for the present study. Abbreviations: LDL-C Low-density lipoprotein cholesterol, MRI Magnetic resonance imaging, VAT Visceral adipose tissue

While a total of 342 adolescents were enrolled in TIGER Kids, the present analytical sample includes 288 adolescents after excluding 54 participants who were missing data for variables necessary for the analyses (Fig. 1). Specifically, participants were missing data for the following: income (*n* = 20), magnetic resonance imaging (MRI; *n* = 11), an insulin value necessary for calculation of the cardiometabolic disease risk score (*n* = 5), a blood sample (*n* = 4), perceived neighborhood disorder (*n* = 2), blood pressure (*n* = 2), race (*n* = 1), or pubertal status (*n* = 1). Additionally, one participant had a triglyceride value above 400 mg/dL which prohibits calculation of low-density lipoprotein cholesterol (LDL-C). Further, seven participants residing in block groups with fewer than 500 residents were excluded as concentrated disadvantage index values for these sparsely populated block groups were unreliable. Participants who were excluded from this study had significantly higher perceived neighborhood disorder, triglycerides, and cardiometabolic risk score values.

### Measurements

#### Neighborhood social environment (chronic stress exposures)

The neighborhood was defined as the block group in which the participant resides. Participants’ home address was geocoded using ArcGIS 10.2 (Environmental Systems Research Institute, Inc., Redlands, CA) and the 2016 U.S. Census TIGER/Line Shapefiles based on the North American Industry Classification. Neighborhood-based chronic stress exposure was operationalized by two neighborhood social environment measures [25]: the neighborhood concentrated disadvantage index and perceived neighborhood disorder.

##### Neighborhood disadvantage

Census data tables used to calculate the neighborhood concentrated disadvantage index were obtained from the 2016 American Community Survey five-year block group estimates, 2012–2016. We followed the PhenX Toolkit protocol for calculation of the neighborhood concentrated disadvantage index originally developed by Sampson, et al. [26] We derived five variables, including percent of the block group that is 1) below the federal poverty line, 2) receiving public assistance, 3) female-headed household, 4) unemployed, and 5) less than age 18 [27]. Each of the five percentage variables were then z-score transformed and summed into a concentrated disadvantage index [28]. A concentrated disadvantage index above 0 indicates a more disadvantaged neighborhood compared to the sample mean while an index value less than 0 indicates a less disadvantaged neighborhood.

##### Perceived neighborhood disorder

Perceived neighborhood disorder was operationalized as adolescents’ perceptions of aesthetic and safety characteristics of their neighborhood which were measured using five and nine items, respectively, from a validated survey [29]. The survey items showed excellent test–retest reliability (86% to 100%) in an Australian sample of 10 year old adolescents [29]. A total perceived neighborhood disorder score was calculated by first reverse scoring the positive neighborhood attributes (e.g., ‘The houses have nice gardens’) so that ‘Yes’ responses indicated the disordered state while ‘No’ responses indicated a lack of disorder. Then, the mean was calculated to create the overall perceived neighborhood disorder score in which a higher score indicates a perception of higher disorder (possible range: 0–1). Four participants were missing a response for one-item each, and their overall scores were calculated from their available responses.

#### Adiposity and cardiometabolic disease risk factors

##### Height, weight, BMI, and waist circumference

Height was measured to the nearest 0.1 cm using a Harpenden stadiometer (Holtain Limited, Crymych, UK). Participants removed shoes, stood upright with heels and back against the stadiometer and head along the Frankfort Plane. Weight was measured to the nearest 0.1 kg using a Michelli GSE 460 scale (G.T. Michelli Co., Baton Rouge, LA) with participants wearing only a hospital gown and undergarments. BMI_p95_ (percentage of the 95^th^ BMI percentile) was calculated based on the participant’s age, height, and weight using the 2000 Centers for Disease Control and Prevention growth charts. BMI_p95_ is the preferred metric for classifying weight status for adolescents with severe obesity as BMI z-scores and percentiles are not strongly related to adiposity measures for BMIs above the 97^th^ percentile [30]. Waist circumference was measured under clothing at the natural waist using a non-elastic tape measure.

##### Body composition imaging

Total body fat mass and region-specific fat deposition (i.e., trunk fat) were assessed by whole-body dual-energy X-ray absorptiometry (DXA) using a GE iDXA scanner (GE Medical Systems, Milwaukee, WI). Visceral abdominal adipose tissue mass (VAT) was assessed by MRI of the abdomen using the General Electric Discovery 750w 3.0 Tesla (GE Medical Systems, Milwaukee, WI). Adipose tissue volumes output by MRI were multiplied by 0.9193 to calculate total mass. For female participants ages 12 and above or females under the age of 12 who were menstruating, a negative urine pregnancy test was required prior to DXA or MRI scans.

##### Blood pressure

Systolic and diastolic blood pressure (SBP and DBP, respectively) were measured using a sphygmomanometer, stethoscope, and appropriately sized arm cuff after the participant had relaxed for five minutes. SBP and DBP were transformed into age-, sex-, and height-specific percentiles specified by the National Heart, Lung, and Blood Institute [31]. Mean arterial pressure was calculated using a standardized formula for inclusion in the continuous cardiometabolic disease risk score [32, 33].

##### Blood chemistry of cardiometabolic disease risk factors

Fasting blood samples were collected by trained phlebotomists using sterile technique. Serum concentrations of insulin, glucose, and blood lipids (i.e., triglycerides and cholesterol) were obtained using a DXC 600 (Beckman Coulter, Inc., Brea, CA). LDL-C was calculated using the Friedewald equation. [34]. Homeostatic Model Assessment for Insulin Resistance (HOMA-IR) was calculated using the formula: fasting insulin (µU/ml) x fasting glucose (nmol/L)/22.5 for inclusion in the continuous cardiometabolic disease risk score [35].

#### Continuous cardiometabolic disease risk score

A continuous cardiometabolic disease risk score was calculated using the five main variables traditionally used to determine metabolic syndrome: 1) waist circumference, 2) high-density lipoprotein cholesterol (HDL-C), 3) triglycerides, 4) mean arterial pressure, and 5) insulin resistance as measured by HOMA-IR [36]. The five risk factors that define the adult metabolic syndrome were chosen to provide consistency for tracking cardiometabolic disease risk across the lifespan [37]. To calculate the score, the five individual risk factors were regressed onto sex, age, pubertal status, and race to account for differences in the risk factors [36]. After obtaining the standardized residuals for each risk factor (HDL-C z-score was multiplied by -1 since it is inversely related to cardiometabolic disease risk), the z-scores were summed to create the cardiometabolic disease risk score such that a higher score indicated a less favorable cardiometabolic disease risk profile [36].

#### Elevated adiposity and cardiometabolic disease risk

Obesity (≥ 95^th^ percentile of age-for-sex BMI) [38, 39], high triglycerides [40], high LDL-C [40], and high blood pressure [31] were classified according to commonly applied definitions for adolescents. High triglycerides were defined as having triglycerides ≥ 90 mg/dL, and LDL-C was considered high if ≥ 130 mg/dL [40]. Participants were considered to have elevated blood pressure if their SBP or DBP was ≥ 90th percentile for sex, age, and height [31]. Sample-specific tertiles of age-adjusted body fat mass and abdominal VAT mass were calculated, and the top tertile was used for analysis.

#### Covariates

Demographic variables (e.g., race, sex, age, annual household income) were measured using a parent-reported survey. Race was collapsed into African American versus Other race (90.3% White). The 18 participants who selected a race other than African American or White were too few to be considered as a separate category; therefore, they were grouped with White participants based on having greater similarities with White participants across other variables. Within each racial group, income was collapsed into tertiles within racial groups as follows: (a) within African American participants: (1) < $50,000, (2) $50,000—$89,999, and (3) ≥ $90,000 per year and (b) within participants of other races: (1) < $90,000, (2) $90,000—$139,999, and (3) ≥ $140,000 per year. These levels were created to address two related issues: (a) statistical model instability as race and income were correlated (*p* < 0.0001) and (b) to ensure the most balanced distribution of income within each racial group as 55.4% of participants of other races and just 13.4% of African American participants lived in households earning $110,000 or more per year. Participants reported their pubertal development using standardized, validated images of stages of development from 1 (no development) to 6 (complete development) for adolescents’ pubic hair development [41]. Pubertal development was collapsed into two categories to reflect (1) pre- or peri-pubertal or (2) post-pubertal.

### Statistical analysis

Demographic and descriptive characteristics were assessed using means and frequencies. Variables with non-normal distributions were subjected to the natural log transformation, and natural log transformed results were back transformed to the original scale for presentation. Statistical analyses were conducted using SAS version 9.4 (Cary, NC), and statistical significance was accepted at *p* < 0.05.

Linear trends among the neighborhood chronic stress exposures (neighborhood disadvantage and perceived disorder), adiposity, and cardiometabolic disease risk factors were tested using random intercept, multilevel linear regression models (PROC MIXED) with participants nested within neighborhood block group. Random intercept models were used to account for correlations among participants living in the same block group (i.e., neighborhood clustering). Analyses were conducted with the neighborhood variables categorized into tertiles representing low, moderate, and high levels of disadvantage and disorder. Conditional means of adiposity and cardiometabolic disease risk measures for each level of neighborhood risk were expressed as least squares means. Tests for linear trends in neighborhood risk were estimated using linear contrasts.

The odds of elevated adiposity and cardiometabolic disease risk associated with neighborhood disadvantage and disorder were assessed using generalized multilevel logistic regression models (PROC GLIMMIX) with a random intercept for block group. All analyses adjusted for age, sex, race, annual household income, and pubertal development status. Degrees of freedom were specified when necessary or calculated using the Kenward-Roger approximation [42].

## Results

Participants were nested within their census block group, with 162 block groups represented in the sample and an average of 1.8 adolescents per block group. The study sample had a mean age of 12.6 ± 1.9 years old, and girls were older than boys, on average (Table 1). Overall, 32% of participants were African American, and 38% reported an annual household income less than $70,000. Mean levels of neighborhood concentrated disadvantage and perceived neighborhood disorder were -0.06 ± 0.6 and 0.19 ± 0.17, respectively. One-third of participants had obesity (e.g., ≥ 95^th^ BMI percentile) and 16.4% had severe obesity (e.g., ≥ 120% of the 95^th^ BMI percentile). Approximately 27% of participants had high triglycerides, 5% had high LDL-C, and 16% had elevated blood pressure.

**Table 1:** Descriptive characteristics of study participants (*N* = 288)

	**Total**
Age (y)	12.6 ± 1.9
Sex	
Male	135 (46.9)
Female	153 (53.1)
Pubertal Stage	
Pre- or Peri-Pubertal	185 (64.2)
Post-Pubertal	103 (35.8)
Race	
African American	93 (32.3)
Other Race	195 (67.7)
Annual Income	
Less than $29,999	30 (10.4)
$30,000-$69,999	78 (27.1)
$70,000-$109,999	58 (20.2)
$110,000-$139,999	50 (17.4)
$140,000 and above	72 (25.0)
Neighborhood Chronic Stress Exposures	
Concentrated Disadvantage Index	-0.06 ± 0.6
Low	93 (32.3)
Moderate	99 (34.4)
High	96 (33.3)
Perceived Neighborhood Disorder	0.19 ± 0.17
Low	111 (38.5)
Moderate	69 (24.0)
High	108 (37.5)
Adiposity and Cardiometabolic Disease Risk Factors	
BMI_p95_ (%)	93.7 ± 27.6
Waist circumference (cm)	78.5 ± 17.8
Total body fat (%)	34.6 ± 10.2
Trunk fat (%)	43.8 ± 7.3
Abdominal VAT mass (kg)	0.55 ± 0.45
LDL-cholesterol (mg/dL)	93.8 ± 21.4
Triglycerides (mg/dL)	74.2 ± 40.4
Systolic blood pressure (%ile)	51.1 ± 26.8
Diastolic blood pressure (%ile)	55.7 ± 25.6
Cardiometabolic disease risk score	-0.19 ± 3.12
Elevated Adiposity and Cardiometabolic Disease Risk	
Top tertile fat mass	96 (33.3)
Top tertile abdominal VAT mass	96 (33.3)
Obese^a^	97 (33.7)
High triglycerides^b^	78 (27.1)
High LDL-C^c^	13 (4.5)
Elevated blood pressure^d^	46 (16.0)

Values are mean ± SD or frequency (%)

*Abbreviations*: *BMI* Body mass index, *BMI*_*p95*_ percentage of the 95^th^ BMI percentile, *VAT* Visceral adipose tissue, *LDL* Low-density lipoprotein cholesterol

^a^Obesity was defined as BMI percentile ≥ 95th percentile for age and sex

^b^High triglycerides was defined as ≥ 90 mg/dL

^c^High LDL-cholesterol was defined as ≥ 130 mg/dL

^d^Elevated blood pressure was defined as a systolic or diastolic blood pressure ≥ 90^th^ percentile for age, sex, and height

Across neighborhood concentrated disadvantage levels, there was a significant *p* for trend for BMI percent of the 95^th^ percentile, waist circumference, total percent body fat, and abdominal VAT mass in that adolescents living in neighborhoods with higher levels of disadvantage had significantly higher BMI, waist circumference, total percent body fat, and abdominal VAT mass than those in less disadvantaged neighborhoods (all *p* for trend < 0.05; Table 2). Association patterns for adiposity linear trends were similar for perceived neighborhood disorder. Across three levels of perceived neighborhood disorder, there was a significant *p* for trend for waist circumference, total percent body fat, and truncal body fat percent (all *p* for trend < 0.05). Adolescents reporting higher levels of perceived neighborhood disorder had significantly higher waist circumference, total percent body fat, and truncal body fat percent, compared with lower disorder neighborhoods. While none of the other markers of cardiometabolic disease risk were statistically significantly related to neighborhood disadvantage, higher levels of perceived neighborhood disorder were associated with higher diastolic blood pressure percentile (*p* = 0.004).

**Table 2:** Least-squares means for adiposity and cardiometabolic disease risk across chronic neighborhood stress exposure levels (*N* = 288)^a^

	Neighborhood Concentrated Disadvantage Index^b^				Perceived Neighborhood Disorder^b^			
	Low	Moderate	High	P for trend^c^	Low	Moderate	High	P for trend^c^
BMI % of the 95^th^ %ile	87.3 (1.0)	92.8 (1.0)	96.2 (1.0)	0.04*	89.4 (1.0)	91.7 (1.0)	95.7 (1.0)	0.06
Waist circumference, cm	74.8 (1.0)	77.8 (1.0)	81.0 (1.0)	0.03*	75.8 (1.0)	78.2 (1.0)	80.2 (1.0)	0.04*
Total fat mass, %	32.7 (1.2)	33.9 (1.2)	36.1 (1.1)	0.04*	32.4 (1.0)	34.5 (1.3)	36.1 (1.0)	0.005**
Trunk fat mass, %	42.8 (0.9)	43.4 (0.9)	45.0 (0.8)	0.07	42.6 (0.7)	44.2 (0.9)	44.8 (0.7)	0.02*
VAT mass, kg	0.36 (1.1)	0.39 (1.1)	0.47 (1.1)	0.04*	0.37 (1.1)	0.42 (1.1)	0.44 (1.1)	0.09
Triglycerides, mg/dL	59.5 (1.1)	65.4 (1.1)	63.4 (1.1)	0.43	60.2 (1.1)	66.3 (1.1)	63.4 (1.1)	0.45
LDL-C, mg/dL	92.8 (2.6)	94.6 (2.5)	89.8 (2.4)	0.40	90.8 (2.3)	94.1 (2.8)	92.6 (2.2)	0.56
Systolic BP, %ile	54.5 (3.0)	48.7 (2.9)	52.1 (2.9)	0.55	51.8 (2.8)	50.1 (3.5)	52.5 (2.8)	0.85
Diastolic BP, %ile	56.7 (3.0)	56.2 (2.9)	56.6 (2.8)	0.97	51.6 (2.7)	54.7 (3.3)	62.1 (2.6)	0.004**
Cardiometabolic disease risk score	-0.57 (0.39)	-0.09 (0.38)	0.04 (0.36)	0.25	-0.62 (0.34)	-0.22 (0.41)	0.25 (0.33)	0.33

Results are shown as least-squares mean estimates (SE). Bold indicates statistical significance, p for trend < 0.05

The following variables were natural logarithm transformed and back transformed for presentation: BMI percent of the 95^th^ percentile, waist circumference, VAT mass, and triglycerides

*Abbreviations*: *BMI* Body mass index, *%* Percent, *%ile* Percentile, *VAT* Visceral abdominal adipose tissue, *LDL-C* Low-density lipoprotein cholesterol, *BP* Blood pressure

^a^Low reflects the lowest level of exposure to chronic neighborhood stress and High reflects the highest exposure

^b^Multi-level models adjusted for age, sex, race, annual household income, pubertal status, and included a random intercept for neighborhood block group

^c^Test for linear trend across the 3 levels of each neighborhood chronic stress exposure measure

^*^p < 0.05; **p < 0.01

**Fig. 2:**
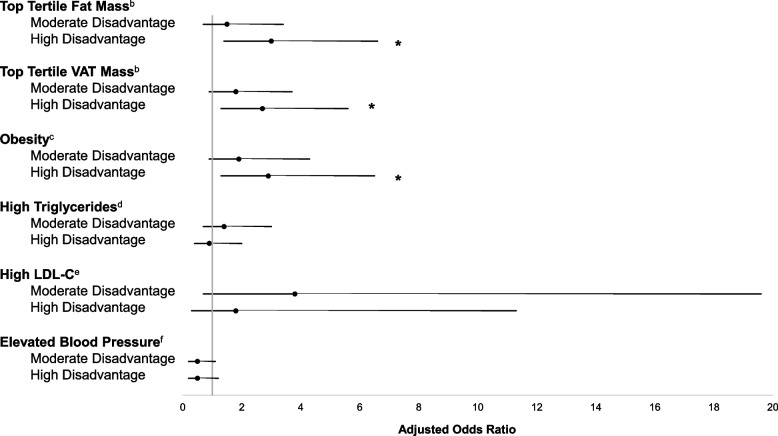
Odds of elevated adiposity and cardiometabolic disease risk for levels of neighborhood concentrated disadvantage.^a^ *Indicates statistical significance at *p* < 0.05. Abbreviations: VAT, visceral abdominal adipose tissue. LDL-C, low-density lipoprotein cholesterol. ^a^*N* = 288. Multi-level models adjusted for age, sex, race, annual household income, pubertal status, and included a random intercept for neighborhood block group. Results shown as adjusted odds ratios. Whiskers show 95% confidence intervals. ^b^Adjusted for age. ^c^Obesity was defined as BMI percentile ≥ 95^th^ percentile for age and sex. ^d^High triglycerides was defined as ≥ 90 mg/dL. ^e^High LDL-C was defined as ≥ 130 mg/dL. ^f^Elevated blood pressure was defined as a systolic or diastolic blood pressure ≥ 90^th^ percentile for age, sex, and height

**Fig. 3:**
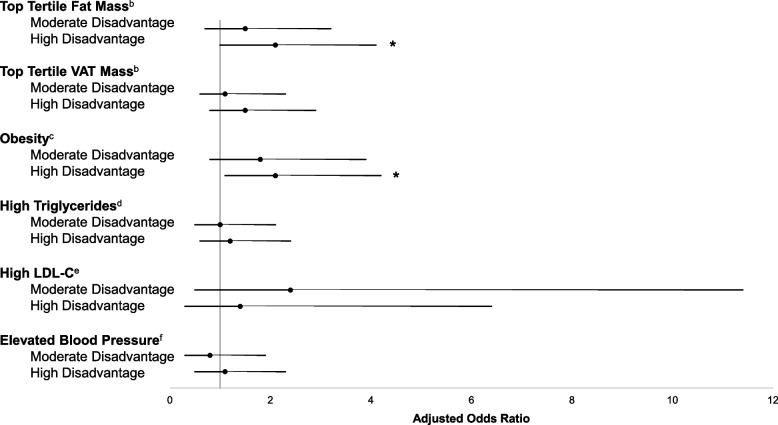
Odds of elevated adiposity and cardiometabolic disease risk for levels of perceived neighborhood disorder.^a^ *Indicates statistical significance at *p* < 0.05. Abbreviations: VAT, visceral abdominal adipose tissue. LDL-C, low-density lipoprotein cholesterol. ^a^*N* = 288. Multi-level models adjusted for age, sex, race, annual household income, pubertal status, and included a random intercept for neighborhood block group. Results shown as adjusted odds ratios. Whiskers show 95% confidence intervals. ^b^Adjusted for age. ^c^Obesity was defined as BMI percentile ≥ 95^th^ percentile for age and sex. ^d^High triglycerides was defined as ≥ 90 mg/dL. ^e^High LDL-C was defined as ≥ 130 mg/dL. ^f^Elevated blood pressure was defined as a systolic or diastolic blood pressure ≥ 90^th^ percentile for age, sex, and height

Logistic regression analyses (Fig. 2) revealed adolescents who resided in the highest disadvantage neighborhoods had 2.9 (95% CI: 1.3, 6.5), 2.7 (95% CI: 1.3, 5.6), and 3.0 (95% CI: 1.4, 6.6) times higher odds of having obesity and elevated (top tertile) abdominal VAT mass and total body fat mass, respectively, compared to those in neighborhoods with the lowest levels of disadvantage (lowest tertile). Similarly, adolescents living in neighborhoods with the highest levels of perceived disorder had 2.1 (95% CI: 1.1, 4.2) and 2.1 (95% CI: 1.04, 4.1) times the odds of having obesity and elevated total body fat mass, respectively (Fig. 3). No statistically significant associations were identified for neighborhoods with moderate levels of disadvantage and disorder. Additionally, moderate and high levels of neighborhood disadvantage and disorder were not statistically significant predictors of elevated lipids or blood pressure when compared to the lowest levels of disadvantage and disorder.

## Discussion

Adolescence is a critical developmental period during which exposure to adverse environments may lead to chronic stress, excess adiposity, and suboptimal cardiometabolic health persisting throughout the lifespan. The present study examined the effects of neighborhood social environment measures of chronic stress exposure on adiposity and cardiometabolic disease risk in adolescence. Neighborhood chronic stress exposure, as measured by neighborhood concentrated disadvantage and perceived neighborhood disorder, was related to adiposity but not most other metrics of cardiometabolic health.

The neighborhood chronic stress measures were categorized into tertiles representing low, moderate, and high levels of disadvantage and disorder to investigate whether there was a linear trend in the relationships in which rising dose/levels of neighborhood-based stress are associated with rising levels of adiposity and cardiometabolic disease risk. Indeed, there was statistically significant evidence of a linear trend in many of the examined relationships. Adolescents residing in neighborhoods with more disadvantage and disorder had significantly higher adiposity compared to their counterparts in less disadvantaged and disordered neighborhoods.

Additionally, adolescents living in neighborhoods with the highest levels of disadvantage and disorder had more than twice the odds of having obesity and being in the top tertile of body fat mass compared to those living in neighborhoods with the lowest disadvantage and disorder. These relationships were not identified for those living in neighborhoods with moderate disadvantage and disorder. These relationships persisted after controlling for traditional risk factors, including age, sex, race, household income, and pubertal development status, which suggests that these neighborhood chronic stress exposures have a distinct relationship with adverse body composition during adolescence. Together, these findings suggest that while greater neighborhood disadvantage and disorder across the spectrum from low to high are related to higher markers of adiposity, perhaps only high ‘doses’ are related to evidence of disease (e.g., obesity) or significantly elevated fat mass during adolescence.

Previous research is in concordance with these findings [43]. A recent review [16] of 21 studies that examined the relationship between the neighborhood social environment and adiposity in childhood and adolescence found that higher adiposity was consistently related to neighborhood socioeconomic disadvantage and social disorder. The authors concluded that neighborhood social deprivation is an important factor in childhood and adolescent adiposity beyond individual-level socioeconomic position. Notably, all studies in their review included only BMI- and anthropometric-based measures of adiposity (e.g., BMI percentile, obesity status, etc.). The authors’ search criteria included terms for adiposity and fat mass, but no studies including these outcomes were identified suggesting a gap in the extant literature which the present study sought to address [16].

Neighborhood chronic stress exposures were generally not associated with other cardiometabolic disease risk factors in the present study. Few studies of the health effects of neighborhood-based chronic stress exposures included cardiometabolic disease risk factors other than those related to weight status or adiposity. Kepper and colleagues [44] did so and reported no statistically significant relationships between neighborhood disadvantage and insulin resistance or inflammation in a sample of prepubertal children. Adverse childhood exposures, such as those stemming from living in disadvantaged and disordered neighborhoods, may become biologically embedded during the critical childhood and adolescent periods setting children on a course toward poor health and premature disease that is not evident until adulthood or a certain threshold exposure level is reached [45]. Indeed, a prospective study of more than 500 adults over 40 years of age found that high social disadvantage during childhood was significantly associated with higher cardiometabolic disease risk and the number of chronic diseases in adulthood [46]. Similarly, an analysis of over 9,000 participants in the National Longitudinal Study of Adolescent to Adult Health found that exposure to neighborhood disadvantage in adolescence was associated with an increased risk of metabolic syndrome in emerging and young adulthood, independent of exposure to neighborhood concentrated disadvantage during later life periods [47]. Finally, Krefman and colleagues [48] compiled data across five cohort studies based in the U.S. and abroad comprising nearly 20,000 participants aged 8 to 55 years to determine whether cardiovascular health declines consistently over the life course or if there are influential age windows, or “change points” during which there is a greater loss of cardiovascular health. Indeed, they found two change points with the largest acceleration of cardiovascular health loss occurring during late adolescence at approximately 17 years of age [48]. Collectively, these results support the model positing that adolescence is a sensitive period of development during which exposures, including living in a disadvantaged neighborhood social environment, have a greater impact on disease risk than they would in later developmental periods. However, these impacts may not be evident during adolescence. Our results are in line with this hypothesis as reflected by the study sample’s relatively healthy cardiometabolic disease risk profile (i.e., lipids, blood pressure, blood pressure, and composite cardiometabolic risk score) despite variability in BMI.

Our findings are consistent with Diez Roux’s schematic framework through which the neighborhood environment contributes to health [11]. Briefly, this framework posits that neighborhood-based residential segregation and resource distribution inequalities interact to produce hazardous environments resulting in stress, maladaptive coping behaviors (i.e., not being physically active due to a lack of neighborhood amenities or safety concerns and poor dietary habits resulting from a lack of nutrient-rich, fresh food options nearby), and poor health. Neighborhoods with these hazardous characteristics are sometimes termed ‘obesogenic environments.’ Obesogenic environments are those with features that promote weight gain and are associated with the development of obesity and poor cardiometabolic health [49]. Such features of obesogenic neighborhoods include those which limit physical activity participation (i.e., a lack of parks, recreation areas, and green space, and low perceived safety) and consumption of healthy diets (i.e., a high density of fast-food restaurants and convenience stores and a low density of supermarkets) [49]. While these features may also be present in low disadvantage neighborhoods, obesogenic environments are more common within disadvantaged neighborhoods including those with low socioeconomic status and high community disadvantage [49].

Additionally, the framework proposed by Diez Roux highlights the need to consider personal characteristics that likely modify these relationships by affecting one’s vulnerability or resources and ability to overcome stressful neighborhood conditions [11]. Although outside of the scope of the current study, future research should explore whether personal characteristics, like sex and race, may moderate the relationships between the neighborhood social environment and cardiometabolic health.

Many studies considering the neighborhood environment have questioned whether objective or perceived measures are more important for various health outcomes. Some studies have found that perceived measures are more strongly related to outcomes such as bicycling, [50] other physical activity behaviors [51], and neighborhood satisfaction [52] while others [49, 53, 54] have failed to find differences between objective and perceived measures. A systematic review of 85 articles involving both adults and children found that perceived measures of the neighborhood environment were more consistently associated with physical activity compared to objective measures although the difference was minimal [51]. Our study found similar associations for perceived and objective neighborhood social environment measures with adolescent adiposity, which is similar to what was found in a study of more than 2,500 adolescents from the Eating and Activity in Teens (EAT) 2010 study. In the EAT study, both low perceptions of safety and greater objectively-measured community disadvantage were related to higher BMI z-scores among boys and girls [49], and the number of police-reported crimes was significantly associated with greater BMI z-score in girls only, whereas perceived crime was positively associated with BMI z-score in both sexes [54]. The neighborhood influence on health outcomes appears to be a complex construct in which both objective and perceived measures are important to consider, especially among children and adolescents.

Our study is not without limitations, including the cross-sectional study design that precludes causal determination. Prospective studies are necessary to disentangle the relationships between neighborhood social environment measures, adiposity, and cardiometabolic disease risk. Additionally, our ability to identify statistically significant relationships for neighborhood disadvantage analyses was limited by the number of block groups in which participants resided as neighborhood disadvantage operates at the neighborhood, not individual, level. Per post-hoc power analyses, we were sufficiently powered at 80% to detect effects of neighborhood disadvantage on waist circumference and body fat measures, but not BMI percentile. Future studies should be powered a priori to detect neighborhood-level and subgroup effects. Lastly, the present study’s results may be affected by misclassification bias in our neighborhood chronic stress measures. We did not assess how long the adolescent lived at their current address thus we did not have the advantage of controlling or stratifying by length of residence. Long-term exposure to neighborhood stressors may be necessary to produce deleterious health outcomes. As such, adolescents who recently moved from lower to higher disadvantage/disorder neighborhoods (or vice-versa) may have been misclassified with respect to their level of neighborhood chronic stress exposure. Future neighborhood-based research should assess and account for length of time at residence as well explore a potential dose–response relationship between the length of residence and health outcomes.

This study has many strengths which warrant discussion. We expand the neighborhood social environment literature by including several factors that recent reviews [16, 25] found lacking in the field. For example, the present study 1) analyzed the neighborhood social environment’s relationship with cardiometabolic disease risk factors and adiposity measures beyond BMI by including advanced imaging measures (e.g., percent body fat mass via DXA, abdominal adipose tissue mass via MRI) finding significant linear trends for many of these advanced measures, 2) calculated BMI percentiles using objectively-measured height and weight rather than relying on self- or parental proxy-report, and 3) utilized published neighborhood definitions (i.e., Census block groups) and validated social environment exposures (i.e., concentrated disadvantage index), which improves confidence in our results and aids in future literature synthesis. Another notable strength of our study is the BMI variability in the TIGER Kids parent study in which nearly one-third of the sample had obesity and 16% had severe obesity. This enables broad generalization of results to adolescents across the BMI spectrum. Finally, our study has made an original contribution by examining relationships among multiple indicators of neighborhood chronic stress exposure and cardiometabolic health. Future research studies should expand on these findings to investigate how these relationships may differ by individual characteristics, such as race and sex, and potential mechanisms through which differences in the health impact of living in stressful neighborhood social environments arise, including stress coping behaviors and the presence or absence of other neighborhood built and social environmental resources.

## Conclusions

This study demonstrates that poor neighborhood social environments, including those with higher neighborhood concentrated disadvantage and perceived disorder, are related to higher adiposity, but not other cardiometabolic risk factors, during adolescence. Findings for both objectively-measured neighborhood concentrated disadvantage and adolescent-perceived neighborhood disorder lend credence to these conclusions. Interventions to address adiposity among adolescents should include measures of the neighborhood environment and be tailored to the individual and their neighborhood to ameliorate the consequences of chronic stress exposure and generate more equitable change.

## Data Availability

The datasets and materials analyzed for the current study are available from the corresponding author on reasonable request.

## Abbreviations

BMI: Body Mass Index
BMI_p95_: Percentage of the 95^th^ Body Mass Index percentil
DBP: Diastolic Blood Pressur
DXA: Dual-Energy X-ray Absorptiometry
EAT: Eating and Activity in Teens
HDL-C: High-Density Lipoprotein Cholesterol
HOMA-IR: Homeostatic Model Assessment for Insulin Resistance
HPA axis: hypothalamic-pituitary-adrenal axi
LDL-C: Low-Density Lipoprotein Cholesterol
MRI: Magnetic Resonance Imaging
REDCap: Research Electronic Data Capture
SBP: Systolic Blood Pressure
TIGER Kids: ranslational Investigation of Growth and Everyday Routines in Kids
U.S.: United States
VAT: Visceral Abdominal Adipose Tissue
